# *CYP9Q1* Modulates Dopamine to Increase Sugar Responsiveness in Honeybees (*Apis mellifera*)

**DOI:** 10.3390/ijms252413550

**Published:** 2024-12-18

**Authors:** Xue-Ling Xu, Long Geng, Zhao-Yang Zeng, Zun Wu, Lin-Feng Li, Shao-Han Tang, Zi-Jing Wang, Han-Hui Shi, Zhi-Guo Li, Hong-Yi Nie, Song-Kun Su

**Affiliations:** College of Bee Science and Biomedicine, Fujian Agriculture and Forestry University, Fuzhou 350002, China; xuelingxu@fafu.edu.cn (X.-L.X.); genglong12022@163.com (L.G.); 18695708867@163.com (Z.-Y.Z.); 15395945898@163.com (Z.W.); 15179757447@163.com (L.-F.L.); tsh1225143858@163.com (S.-H.T.); 17769939857@163.com (H.-H.S.); zhiguo.li@fafu.edu.cn (Z.-G.L.); hnhynie@126.com (H.-Y.N.)

**Keywords:** honeybees, food wanting, dopamine, RNA-seq, *Cytochrome P450*

## Abstract

The appetite of honeybees for food is crucial to their survival and reproduction, as they sustain their entire colony by collecting pollen and nectar for nutrients. Dopamine, an important neurotransmitter, regulates appetite and satiety. However, how dopamine regulates honeybee foraging behavior remains unexplored. In this study, we investigated dopamine expression in 23-day-old *Apis mellifera* under different food-wanting conditions and identified differentially expressed genes (DEGs) in the brains of honeybees using RNA sequencing technology. We showed that dopamine levels in honeybees starved for 2 h were higher than those sated after 2 h of starvation. RNA-seq results revealed there were differences in the expression of cytochrome P450-dependent monooxygenase (*CYP9Q1*) in honeybees, which regulated the sucrose sensitivity of honeybees under different intake states. Furthermore, *CYP9Q1* targeted the expression of the *insulin receptor substrate (IRS)* to promote dopamine synthesis. Our findings emphasize the relationship between dopamine and honeybees’ desire for food at the molecular level, providing a reference for further exploring the mechanism of food wanting.

## 1. Introduction

The honeybee (*Apis mellifera*) is a eusocial insect and an important model organism for studying the mechanisms and evolution of social behaviors [1,2]. The adaptive control of foraging decisions is critical for survival and reproduction and is mediated by complex brain mechanisms [3]. For example, in hungry animals, foraging behaviors can be regulated by a variety of nervous system processes, including those responsible for receiving and processing sensory properties and assigning food rewards [4,5]. At present, elucidating the molecular mechanism of bee foraging behavior remains challenging.

Behavioral changes are associated with gene expression changes in the honeybee brain [6,7]. Dopamine, a key neurotransmitter, is involved in motor control, reward mechanisms, behavioral reinforcement, and specific higher cognitive functions [8,9,10,11]. The level of dopamine in the honeybee brain is directly related to the level of food wanting. When bees are hungry, their appetite increases and the dopamine-dependent wanting system is activated [12]. Conversely, when bees are satiated, dopamine levels decrease, reducing their desire for food [12,13]. Previous studies in our lab have found that dopamine levels in the brains of bees rise significantly, as they route to targets and during waggle dances [12]. This mechanism helps bees find and utilize food resources efficiently in complex natural environments.

Honeybees are social insects that live in colonies, with a reproductive queen and thousands of largely sterile female helpers, called worker bees [14,15]. Worker bees exhibit an age-dependent division of labor, culminating in foraging activities when the bees are between 2 and 3 weeks old [16]. The behavioral states of worker bees depend on their sense of the environment and chemical communication through their olfactory systems [6,17]. Honeybees use olfactory machinery to collect nectar and pollen, linking sugar stimulation to food rewards [18,19]. Dopamine is a critical neurotransmitter that regulates behavior and plays an important role in the brain and olfactory learning behaviors [20,21,22]. The expression level of *Apis mellifera* invertebrate-type dopamine receptors in the brain increased with the age of worker bees [23]. *Tyrosine hydroxylase* (*TH*) is the rate-limiting enzyme in the production of dopamine [24,25]. TH converts tyrosine into L-3,4-dihydroxyphenylalanine (L-DOPA), which is subsequently converted into dopamine [26]. The *insulin receptor substrate* (*IRS*) gene is essential for the transduction of insulin/insulin-like signaling (IIS) [27]. Like vertebrates and other insects, IIS is considered an important integrator of nutrient storage, metabolism, and behavior in honeybees [28,29]. The various components of the IIS pathway respond to dietary manipulation, influence sucrose perception, and are associated with foraging behavior [29].

Currently, there are relatively few studies on the relationship between dopamine and food wanting in honeybees, and the molecular mechanism regulating bee food wanting remains unclear. In the present study, we aimed to determine the genes associated with food wanting. We examined dopamine expression in 23-day-old *Apis mellifera* under different food-wanting conditions and performed RNA-seq on the brains of honeybees that were starved for 2 h and those that were sated after 2 h of starvation.

## 2. Results

### 2.1. Detection of Dopamine in the Brains of Honeybees Under Different Starvation Conditions

**Figure 1:**
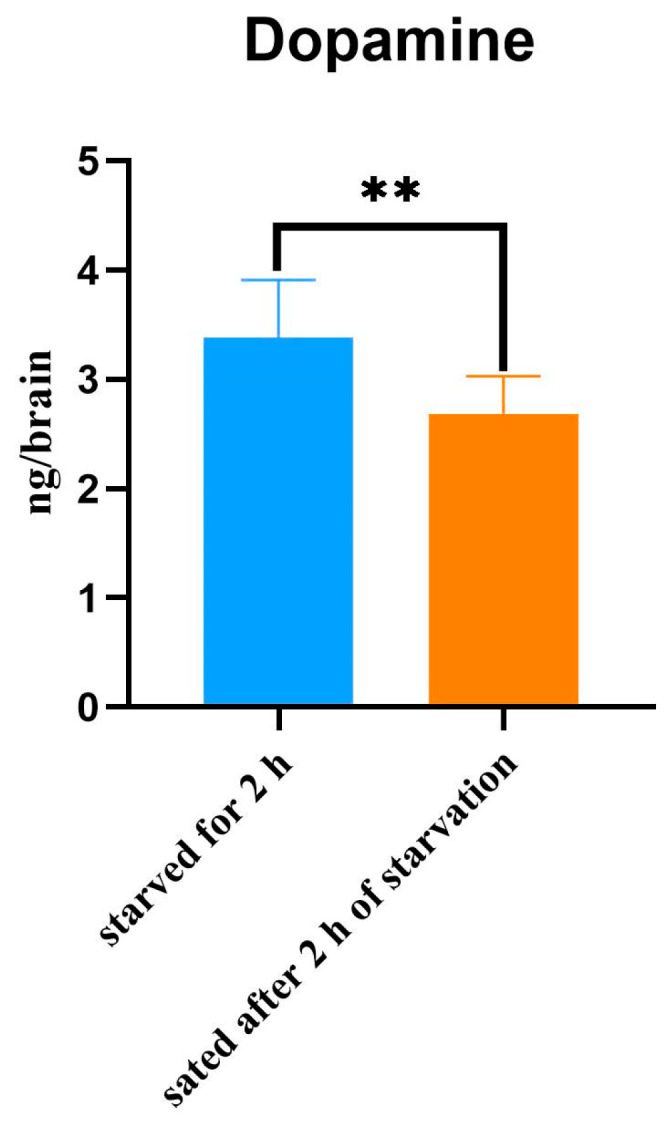
Expression levels of dopamine in brain of honeybees with different starvation conditions. Data were obtained from 10 individuals in each group (** *p* < 0.01).

Dopamine levels in the brains of honeybees with normal proboscis extension responses (PERs) were increased following starvation. The dopamine levels in the brains of honeybees starved for 2 h were significantly higher than those of bees sated after 2 h of starvation (Figure 1). Comparing the changes in dopamine levels in the brains of bees under the two starvation states revealed that the dopamine levels in the brains of bees in the starved state were significantly higher than those in the satiated state, indicating that dopamine is a neurotransmitter related to reward and motivation.

### 2.2. Sequencing Analysis and Quality Control

Through high-throughput sequencing, six libraries yielded a total of 259 million original sequences, with the highest number from sample F2-2 and the lowest from sample H2-3 (Table 1). After filtering low-quality reads, the proportion of effective data was between 97.91% and 98.85%, which reached the necessary detection standards. After cleaning and quality checks, the percentages of Q20 and Q30 were as high as 97.64% and 93.31%, respectively (Table 1). These results indicate that the filtered data were reliable and could be used for subsequent biostatistical analysis.

### 2.3. Real-Time Quantitative PCR (qPCR) of Differentially Expressed Genes (DEGs)

**Figure 2:**
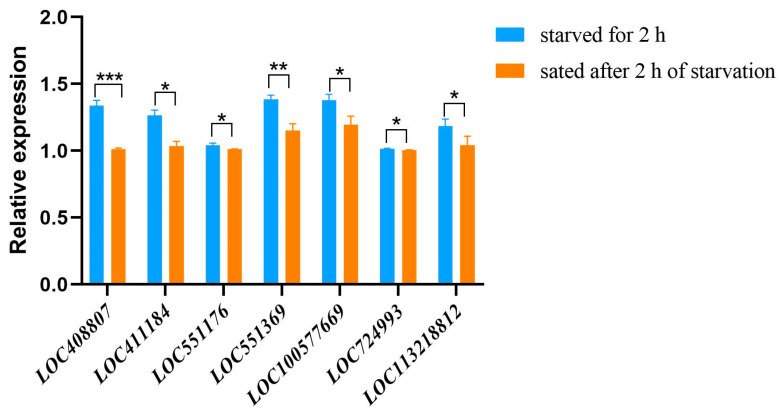
Real-time quantitative PCR (qPCR) analysis of differentially expressed genes (DEGs) between bees starved for 2 h and bees sated after 2 h of starvation (* *p* < 0.05; ** *p* < 0.01; *** *p* < 0.001).

In order to verify the accuracy of RNA-seq results, seven DEGs were selected from the transcriptome and verified using real-time fluorescent qPCR. As shown in Figure 2, the expression levels of the selected seven DEGs detected in the starved state of bees were higher than those in the satiated state after 2 h of starvation. This trend was consistent with the transcriptome trend, reflecting the reliability of RNA-seq.

### 2.4. Analysis of Differentially Expressed Genes (DEGs)

**Figure 3:**
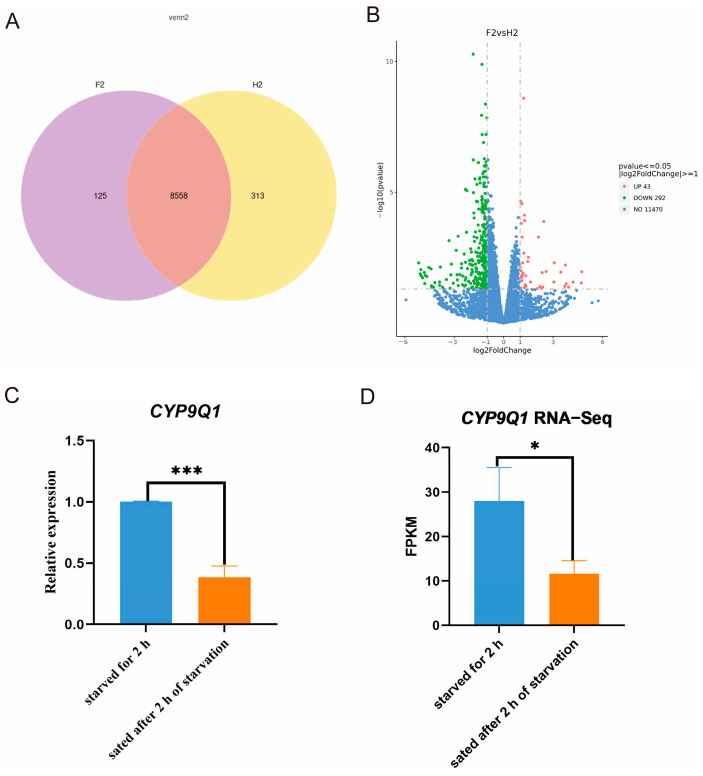
Differential gene expression between in different starvation conditions of honeybees was analyzed. (**A**) Differential gene clustering plot. (**B**) Volcano plot for differentially expressed genes (DEGs) between starved for 2 h and sated after 2 h of starvation groups. Each point in the volcanic map represents a protein. (**C**) cytochrome P450-dependent monooxygenase (*CYP9Q1*) mRNA levels quantified by qPCR (*** *p* < 0.001). (**D**) *CYP9Q1* mRNA levels in experimental and control groups from transcriptome sequencing (* *p* < 0.05).

There were 8558 genes co-expressed in the starvation and satiety states of bees, of which 125 genes were uniquely expressed in the satiated state after 2 h of starvation, and 313 genes were uniquely expressed in the starved state after 2 h (Figure 3). According to the differential gene volcanogram, it was found that 293 DEGs were upregulated, and 43 DEGs were downregulated after 2 h of starvation compared with satiation after 2 h of starvation (Figure 3 and Figure S1).

The genes with a high expression of *p* < 0.05, log2Fold > 1, and log2Fold < −1 were screened from the differential genes. The main differential genes were focused on metabolic product synthesis, metabolic processes, neural development, immune function, muscle contraction, and motor coordination processes. These differential genes reflect how bees regulate their physiology and behavior to adapt to environmental pressures under different starvation conditions. Furthermore, we found that the expression of cytochrome P450-dependent monooxygenase (*CYP9Q1*) in bees starved for 2 h was significantly higher than in bees sated after 2 h of starvation (Figure 3C,D and Figure S1). Cytochrome P450 is involved in metabolism, detoxification, and hormone synthesis in honeybees. Dopamine is produced by the decarboxylation of tyrosine to tyramine, followed by the hydroxylation of the benzene ring by cytochrome P450. Together, these findings indicate that *CYP9Q1* is associated with dopamine production and is required for driving food-wanting behaviors under different hunger states.

### 2.5. GO and KEEG Enrichment in Honeybees Under Different Food Desire States

**Figure 4:**
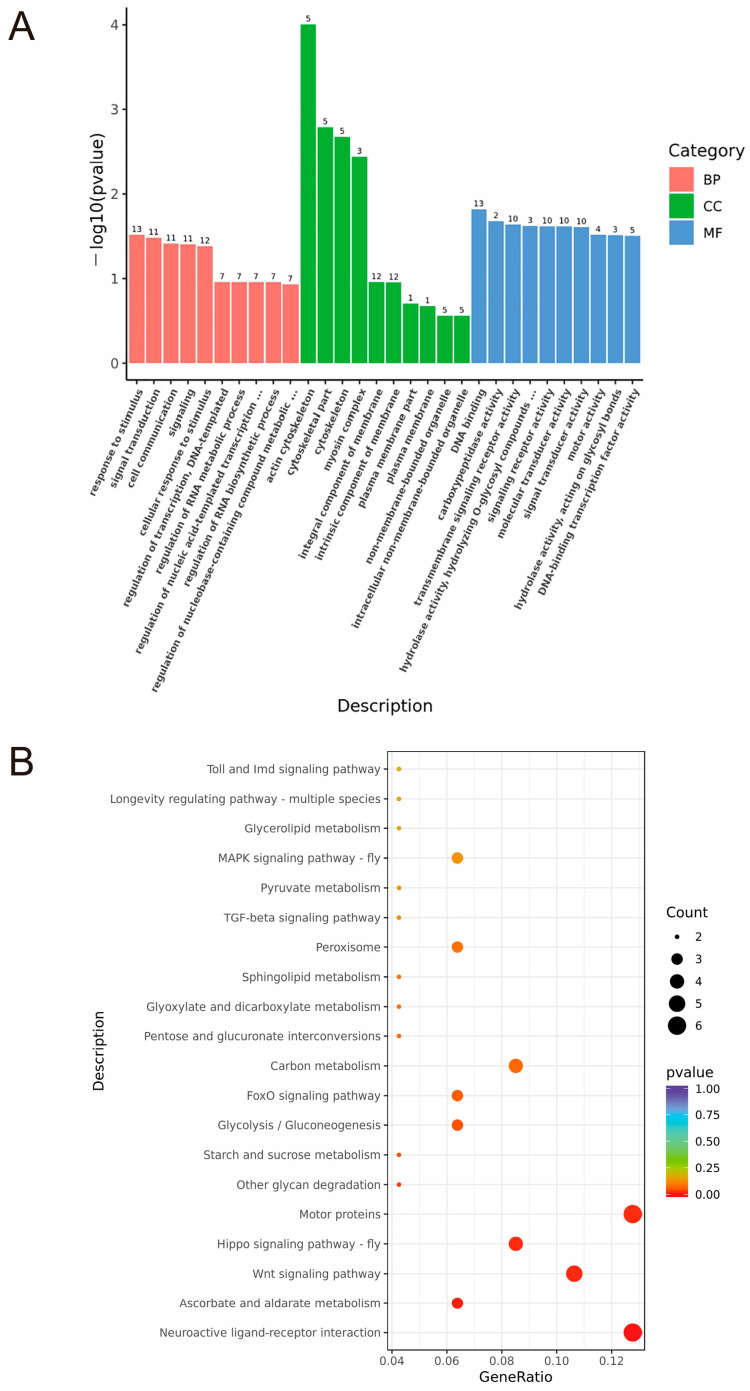
(**A**) Gene Ontology (GO) enrichment analysis of honeybees in different food desire states. The top 10 significantly enriched GO terms of the differentially expressed proteins (DEPs) in biological process (BP), molecular function (MF), and cellular component (CC). (**B**) Analysis of Kyoto Encyclopedia of Genes and Genomes (KEGG) pathways in honeybees under different food desire states.

A total of 335 DEGs were screened from the brains of honeybees under different starvation conditions and further analyzed using Gene Ontology (GO) enrichment analysis. The results showed that 180 DEGs of these differential genes were annotated in 258 GO terms, of which 59 differential genes involved in molecular function (MF) were annotated in 134 GO terms, 29 differential genes involved in cellular components (CCs) were annotated in 21 GO terms, and 92 differential genes involved in biological processes (BPs) were annotated in 103 GO terms (Figure 4A and Figure S2).

The differentially expressed proteins (DEPs) were classified via GO annotation based on three categories: BP, MF, and CC (Figure 4A and Figure S2). Of the BP terms, stress responses, signal transduction, cellular communication, and cellular responses to stimuli were all enriched, which is related to how bees respond to changes in their environment or internal state. DEPs in CCs are mainly concentrated in the actin cytoskeleton, cytoskeleton, and myosin complex. Different food desire states may alter the function or expression of these structural components. The GO channels of MF mainly focus on enzyme activity, transmembrane signal receptor activity, signal receptor activity, and binding transcription factor activity, which are related to signal transduction and regulation mechanisms inside and outside the cells of honeybees.

To determine the potential pathways of differential genes for bees starved for 2 h and sated after 2 h of starvation, KEGG pathway analysis was performed. Forty-seven genes were successfully annotated into 51 pathways (Figure 4B and Figure S2). These important pathways are mainly enriched in neuroactive ligand–receptor interactions, glycolysis/gluconeogenesis, starch and sucrose metabolism, and ascorbic acid and Arabic acid metabolism.

### 2.6. Inhibition of CYP9Q1 in Honeybees

**Figure 5:**
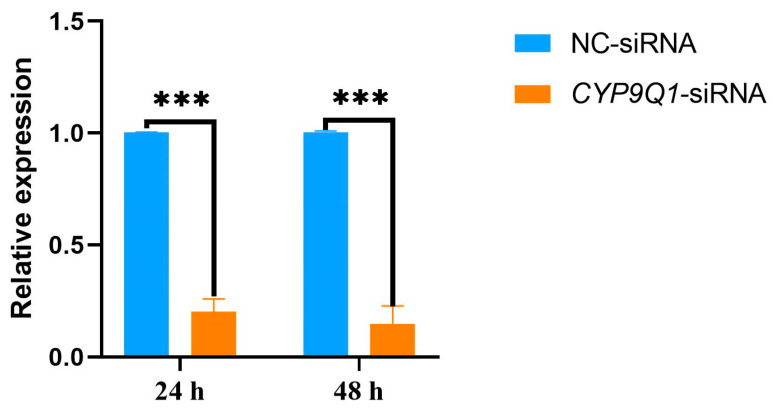
*CYP9Q1* expression in the brains of honeybees after oral feeding with *CYP9Q1*-siRNA or negative control nonsense sequences (NC-siRNA; *** *p* < 0.001.).

Considering the importance of *CYP9Q1* in behavioral transitions, we decided to inhibit its expression in honeybees to examine the possible effects on behavior. Synthetic siRNA was fed to bees tested for sugar water sensitivity in sucrose solution. qPCR confirmed the inhibition of *CYP9Q1* in the brain of honeybees 24 h and 48 h after siRNA feeding. Our results showed that *CYP9Q1* expression in the brains of honeybees from the *CYP9Q1*-siRNA group was significantly lower than in the NC-siRNA group (*p* < 0.001) (Figure 5).

### 2.7. CYP9Q1 Affects Honeybees’ Consumption of Sugar Water

**Figure 6:**
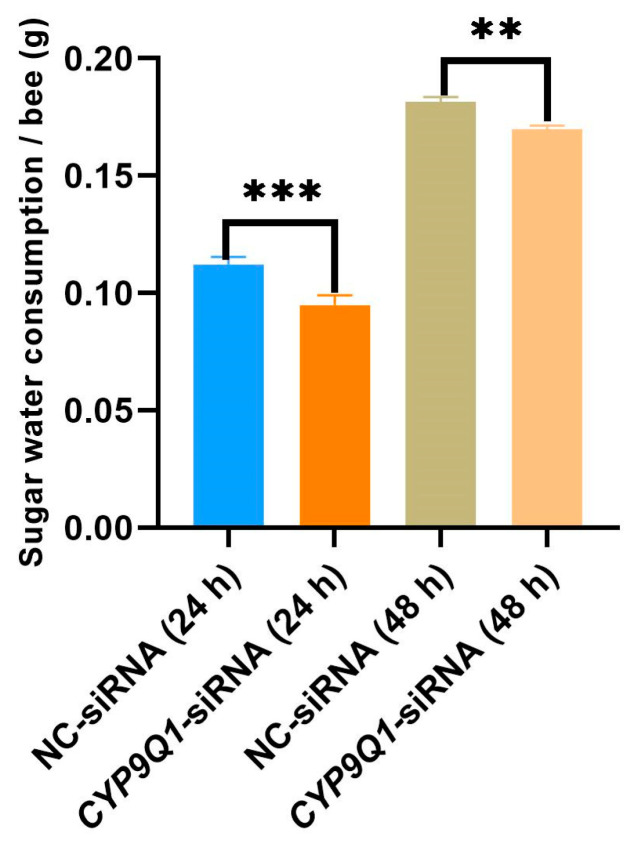
The effect of *CYP9Q1* on the sugar water consumption of honeybees. Sugar water consumption in honeybees treated with small interfering RNA of *CYP9Q1* and nonsense sequence (** *p* < 0.01; *** *p* < 0.001).

To further investigate the possible function of *CYP9Q1* in honeybees’ sucrose responsiveness, we tested the effect of *CYP9Q1* on sugar water consumption through siRNA. Sugar water consumption varied significantly over time, increasing with the duration of starvation. Bees fed with *CYP9Q1*-siRNA showed significantly lower sugar water consumption compared to control bees fed with nonsense control (NC-siRNA) (Figure 6). These results suggest that the *CYP9Q1* gene is closely related to the food appetite of bees. Inhibiting the *CYP9Q1* gene may affect the metabolic capacity of bees, change their way of processing sugars, and consequently, lead to a decrease in the intake of sugar water.

### 2.8. CYP9Q1 Affects Honeybees’ Sensitivity to Sugar Water

**Figure 7:**
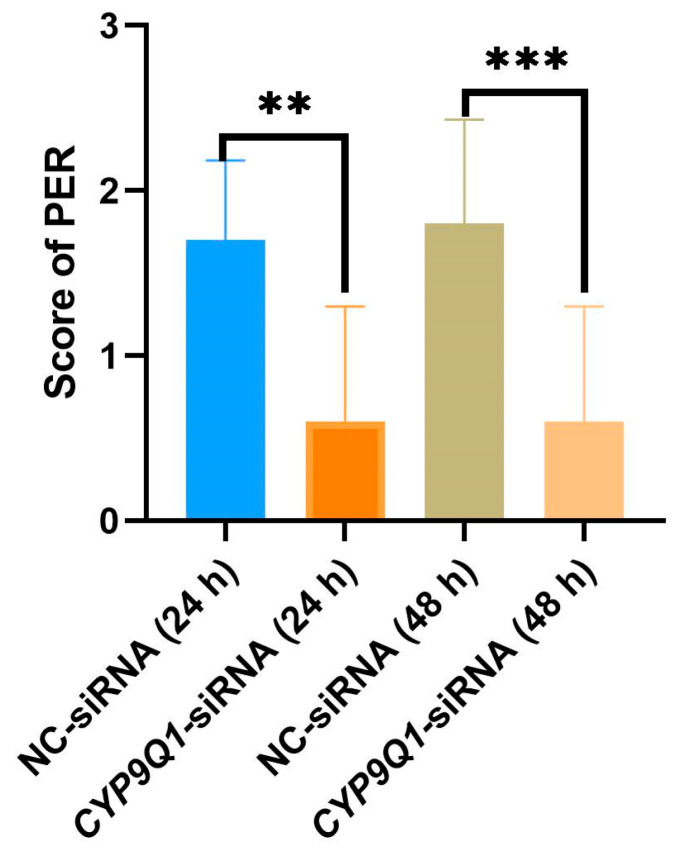
The effect of *CYP9Q1* on the sugar water sensitivity of honeybees. (**A**) Score of proboscis extension response (PER) assay. (**B**) Honeybees were treated with the small interfering RNA of *CYP9Q1* and nonsense sequence at 24 h and 48 h; then, the sugar water sensitivity of bees was measured by PER assay (** *p* < 0.01; *** *p* < 0.001).

To further investigate the possible function of *CYP9Q1* in the honeybee behavioral transition, we tested the effect of *CYP9Q1* on PER through siRNA. Ten bees in different states were selected from the constant temperature incubator. After 2 h of starvation, the bees were tested for sugar water sensitivity and scored. Bees fed with *CYP9Q1*-siRNA showed significantly lower PER responses compared to control bees fed with NC-siRNA (Figure 7). Moreover, the longer the incubation time after interference, the lower the sensitivity. It is inferred that interfering with the expression of the bee *CYP9Q1* gene may affect metabolic function. These enzymes are usually involved in poison metabolism and drug detoxification. The reduced sensitivity to sugar water may be due to changes in these enzymes that affect the ability of bees to process sugar or their energy metabolism.

### 2.9. CYP9Q1 Regulates the Expression of TH and IRS

**Figure 8:**
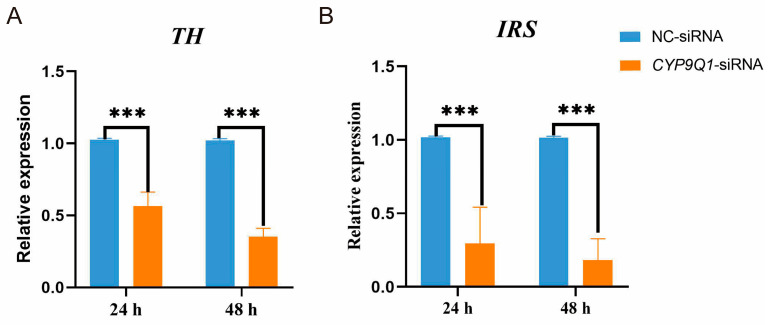
Relative expressive levels of (**A**) *tyrosine hydroxylase* (*TH*) and (**B**) *insulin receptor substrate* (*IRS*) in the brains of bees from negative control small interfering RNA (NC-siRNA) and *CYP9Q1*-siRNA groups (*** *p* < 0.001).

To further reveal whether *CYP9Q1* affects dopamine expression and, consequently, bee feeding, we tested the effect of *CYP9Q1* inhibition on the rate-limiting enzyme of dopamine synthesis. We confirmed that siRNA-induced *CYP9Q1* knockdown decreased levels of the TH gene, suggesting that *CYP9Q1* is associated with the synthesis of dopamine in bees (Figure 8A). The IRS is an adapter protein that plays an important role in regulating metabolism, growth, and development. To determine whether *CYP9Q1* affects the *IRS*, we used a real-time PCR assay and found that siRNA-mediated *CYP9Q1* knockdown decreased the expression of *IRS* (Figure 8B). Our data, therefore, suggest that *CYP9Q1* may promote food wanting through the *IRS*.

## 3. Discussion

In conclusion, we have shown that dopamine appears to be the motivator for bees’ food wanting during hunger. Specifically, dopamine is activated during energy deficit to promote food motivation that drives and maintains food-wanting behavior, which is necessary for hunger-triggered food consumption. *CYP9Q1* is a key gene that affects dopamine expression to regulate food motivation for food wanting. Additionally, *CYP9Q1* may affect dopamine expression by regulating the *TH* and *IRS* genes.

Dopamine is a biogenic amine that plays multiple roles in the regulation of behavior and reproduction in eusocial insects [30,31]. For instance, *Apis mellifera* brain dopamine levels briefly increase when bees arrive at a rewarding food source or begin to waggle and dance to recruit nestmates to feed [12,32]. Dopamine affects dance-following behavior and the use of information about high-quality food sources in honeybee foragers [33]. In addition, dopamine promotes food wanting under the conditions of energy deficit. By isolating a single bee in the laboratory and starving it, bees were shown to have significantly increased sucrose sensitivity to layers of sugar water under starvation, as well as significantly increased dopamine levels in the brain. When bees are hungry, they need to search for food. This behavior activates the reward system in their brains, leading to an increase in dopamine levels and enhancing their motivation and efficiency in foraging for food [34]. Therefore, the state of hunger stimulates bees to engage in foraging activities more actively. Hunger is also a physiological stress state. Stress usually causes changes in the dopamine levels within the body, which may result in an elevation of dopamine levels to improve the alertness and mobility of animals under stressful circumstances [35,36]. Resource-seeking behaviors are ordinarily constrained by physiological needs and threats of danger, as is the case in other animals [37,38,39]. *Drosophila* still seek rewards triggered by dopaminergic neural mechanisms in response to electric shock punishment [39]. Additionally, to enhance foraging efficiency and motivation, the brains of bees will release more dopamine [40]. This mechanism helps bees to locate resources more effectively when food is scarce. Thus, food wanting is not just essential for maintaining proper nutrition for the individual but is also an important component in the regulation of the food resources and survival of the colony.

Subsequently, we conducted RNA sequencing tests to examine the DEGs and enrichment pathways between bees starved for 2 h and those sated after 2 h of starvation. Transcriptome results showed that the main differential genes were concentrated in metabolite synthesis, metabolic processes, neurodevelopment, immune function, muscle contraction, and motor coordination processes, indicating that the physiological adaptation mechanism of honeybees is very complex, involving metabolic, neural, immune, and other aspects to ensure their survival and efficiency. This adaptation mechanism helps honeybees optimize energy utilization and maintain physiological functions in the context of limited resources. KEGG analysis was performed on the DEGs of bees that were starved for 2 h and bees fed to satiation after being starved for 2 h. It was found that a total of 47 genes were annotated to 51 pathways, among which the neuroactive ligand–receptor interaction, glycolysis/gluconeogenesis, starch and sucrose metabolism, and ascorbate and aldarate metabolism were significantly enriched. The significant enrichment of these pathways reveals how bees adjust their neural, metabolic, and antioxidant mechanisms to adapt to environmental and internal demand changes under different states of food desire. *CYP* is expressed in specific brain regions and nerve cell types and may be involved in regulating the neurotransmitter metabolism [41,42,43]. In this study, we found a higher level of expression in *CYP9Q1* in bees starved for 2 h, which is consistent with dopamine expression levels. Furthermore, bees fed with *CYP9Q1*-siRNA showed significantly lower sugar water consumption and PER responses. Additionally, the cytochrome P450 mediated the synthesis of dopamine from tyramine, in which TH catalyzes the rate-limiting step. In our study, TH had significantly lower expression in the *CYP9Q1*-siRNA bees compared to the NC-siRNA bees. Overall, our findings confirm that *CYP9Q1* might play a critical role in regulating food wanting and is probably responsible for dopamine synthesis.

The present study also reveals a possible mechanism of how *CYP9Q1* regulates food-wanting behavior. Insulin-like peptide signaling is upstream of dopamine production, and dopamine levels are reduced by knocking out the insulin-like peptide receptor gene (dlnR) in *Drosophila* [44,45]. The inhibition of the *IRS* plays a role in post-insulin receptor signal transduction, affecting honeybee load on nectar and pollen [46]. By reducing the expression of the *IRS* gene via RNA interference, worker foraging behavior is regulated [47]. The bumble bee (*B. ignitus*) *insulin-like peptide receptor gene* (*ILPR*), which encodes insulin-like peptide receptors, is involved in sugar-sensing [48]. Significantly, we found that the expression of the IRS in the *CYP9Q1*-siRNA group was significantly lower than in the NC-siRNA group. This finding indicates that *CYP9Q1* inhibition can decrease the expression of the IRS in the honeybee brain. This suggests that *CYP9Q1* may influence the IRS to regulate dopamine and further contribute to bee-feeding behavior.

Altogether, these findings provide insights into the role of dopamine in honeybee food wanting, showing that the levels of dopamine and *CYP9Q1* gene expression are tightly correlated. The dopamine levels were significantly higher in the brains of bees starved for 2 h than in those sated after 2 h of starvation. The RNAi knockdown of *CYP9Q1* significantly inhibited *CYP9Q1* expression in the brains of bees, resulting in decreased sucrose responsiveness. Furthermore, we found that *CYP9Q1* affects the expression of the *IRS* and *TH* to regulate dopamine synthesis. These findings suggest that *CYP9Q1* may target the dopamine synthesis pathway and, thus, play an important role in regulating honeybee feeding behavior. Further studies are required to verify the exact relationship between *CYP9Q1* and dopamine at the protein and cellular levels.

## 4. Materials and Methods

### 4.1. Honeybee Husbandry

The honeybees (*Apis mellifera*) used in this experiment were selected from the bee species at “Bee Qiang No. 1” in Fuqing Bee Farm and raised at Fujian Agriculture and Forestry University (FAFU), Fuzhou, China. All honeybees used in the experiment were 23-day-old worker bees, because the dopamine content in the brain of foraging bees (generally >3 weeks old) is significantly higher than in young worker bees performing tasks within the colony [7].

Bees were obtained from brood frames taken from the experimental hives and kept in an incubator at 34 °C, with a relative humidity of 50% to 55%. Individual identification was ensured by marking the thorax of the bees with color marks. The marked bees were transferred into the original colony and raised for 23 days.

### 4.2. Honeybee Labeling

When the marked 23-day-old bees were captured, they were transported to the laboratory. They were briefly cooled, anesthetized, and harnessed in a copper tube, allowing for free movement of the antennae and mouthparts. To ensure individual responsiveness to sucrose was normal, bees that showed a clear proboscis extension when the antennae were stimulated with a 50% sucrose solution were used in the experiments. After recovery, the fixed bees were fed 5 μL of a 50% (*w*/*v*) sucrose solution, alternating with ultrapure water, and placed in a dark box maintained at a constant temperature (25 ± 1 °C) and relative humidity (60 ± 2%) to ensure consistent conditions. The bees were divided into 2 groups, each with 10 bees. The control group and experimental groups were formed as follows: bees starved for 2 h and bees sated after 2 h of starvation.

### 4.3. Dissection of Honeybee Brains

The brains of bees starved for 2 h and sated after 2 h of starvation were collected. The collected brains were treated under a dissecting microscope on ice, and then, two compound eyes, three simple eyes, hypopharyngeal glands, tracheae, and further glandular tissues were carefully removed. Dissected brains (10 brains per group) were collected into an empty 1.5 mL centrifuge tube and stored individually at −80 °C until analysis.

### 4.4. High-Performance Liquid Chromatography (HPLC) Analyses of Dopamine Levels in the Honeybee Brain

Brains (10 brains per group) were taken out of the −80 °C freezer and homogenized using a high-flux tissue homogenizer. Dopamine levels were measured using an Acquity High-Performance Liquid Chromatograph (Waters, Milford, MA, USA). The column temperature was maintained at 40 °C, and the mobile phase flow rate was set at 0.25 mL/min. Detection was performed at 350 mV. Two measurements were taken from each sample, and ten samples were tested for each treatment. Dopamine was quantified from single-brain tissue samples.

By pipetting the appropriate dopamine standard stock solution and diluting it to 100 ng/mL, 80 ng/mL, 60 ng/mL, 40 ng/mL, 20 ng/mL, 10 ng/mL, and 5 ng/mL, the solutions were sequentially analyzed using the instrument. The linear regression equation was fitted as y = 1.76e + 06x + 1.38e + 06. Through the detection of the dopamine standard using HPLC-ECD, the retention time of the dopamine standard was found to be 5.153 min, confirming that the peak appearing around this time period represents dopamine in the bee brain samples (Supplementary Figure S3).

### 4.5. Library Preparation and RNA Sequencing

According to the manufacturer’s protocol, total RNA was extracted from bee brains (10 bees per group) using the TRIzol kit (Invitrogen, Carlsbad, CA, USA). The extracted total RNA samples were evaluated as follows: (1) 1% agarose gel electrophoresis was used to assess RNA integrity and contamination; (2) RNA purity was measured using a NanoDrop spectrophotometer (Thermo Fisher Scientific, Waltham, MA, USA); (3) total RNA concentration was determined using a Qubit^®^ 2.0 Fluorometer (Life Technologies, Carlsbad, CA, USA); and (4) RNA integrity was assessed using an Agilent 2100 (Agilent, Santa Clara, CA, USA) for library construction. After total RNA extraction, eukaryotic mRNA was enriched with Oligo (dT) beads. The enriched mRNA was then fragmented with fragment buffer and reverse-transcribed to cDNA using the NEBNext Ultra RNA Library Prep Kit for Illumina (neb #7530, New England Biolabs, Ipswich, MA, USA). End repair was performed on the purified double-stranded cDNA fragment, bases were added, and the Illumina sequencing adapter was attached. To select cDNA fragments from 150 to 200 bp, the library fragments were purified using the AMPure XP system (Beckman Coulter, Beverly, CA, USA). The size of the linked fragments was selected via agarose gel electrophoresis and amplified using the polymerase chain reaction. The cDNA library was sequenced using the Illumina HiSeq^TM^ 4000 system by Novogene (Beijing, China).

According to the read counts, the reference genomes were compared using HISAT2 software (version 2.1.0), the transcripts were reconstructed with StringTie, and the expression values (FPKM) of all genes in each sample were calculated using RNA-Seq by Expectation-Maximization (RSEM). The FPKM value was used as the gene expression indicator, and the differential expression was analyzed with DESeq2 software (version 1.26.0). DEGs were screened according to the significant differential expression threshold (*p*-value < 0.05). GO enrichment and pathway enrichment analyses of DEGs were performed using the ClusterProfiler R software package (version 4.1.0), combined with the GO enrichment and KEGG databases, with *p* < 0.05 as the significance threshold for enrichment. Differentially expressed mRNA genes were randomly selected for real-time qPCR verification. The gene primers are provided in Supplementary Table S1.

### 4.6. siRNA Preparation and Feeding

*CYP9Q1*-specific small interfering RNA (siRNA) (sense: 5′-CAGAUUGUUCAAUUUGAAGTT-3′, antisense: 5′-CUUCAAAUUGAACAAUCUGTT-3′) was designed and synthesized by Shenggong Bioengineering Co., Ltd. (Shanghai, China). During siRNA feeding, 10 µg of *CYP9Q1*-siRNA solution was fed to each bee that had been starved for 2 h, and the same dose of NC-siRNA solution was fed to the control group. All honeybees were then incubated in an incubator (temperature: 34 ± 1 °C, relative humidity: 75 ± 2%) for 24 h or 48 h, with 10 bees in the experimental group and 10 bees in the control group under each culture condition. The honeybees’ sugar water consumption was recorded during the experiment, and the sugar water sensitivity was tested. After the experiment, the honeybee brains were dissected, knockout efficiency was verified using real-time qPCR, and β-actin was used as the internal reference gene.

### 4.7. Testing Sensitivity to Sugar Water

**Figure 9:**
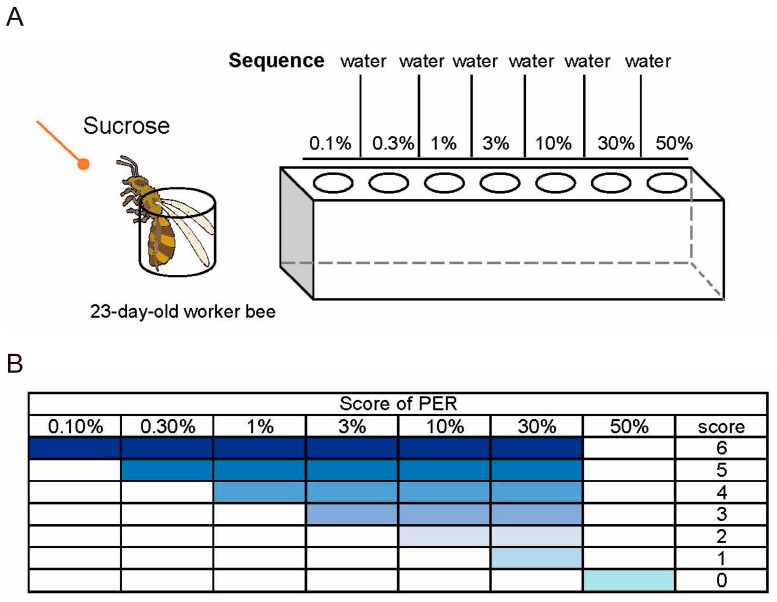
Testing responsiveness to sugars. (**A**) Flow chart of the sucrose solution sensitivity experiment. (**B**) Score of proboscis extension response (PER) assay.

The experimental and control groups (N = 10) under different culture conditions were tested for sugar water sensitivity. Before the experiment, sucrose solutions of 0.1%, 0.3%, 1%, 3%, 10%, 30%, and 50% (*w*/*v*) were prepared [49,50]. Before each sugar concentration test, the responsiveness of each bee to water was tested to prevent sensitization. Bees were mounted using wooden toothpicks dipped in sugar water or ultrapure water in the following order: 0.1%, ultrapure water; 0.3%, ultrapure water; 1%, ultrapure water; 3%, ultrapure water; 10%, ultrapure water; 30%, ultrapure water; and 50%, to carry out sugar water sensitivity tests (Figure 9A). The responses to all seven concentrations of sugar constituted a PER score for each bee (Figure 9B).

### 4.8. Quantification of Transcript Expression After siRNA Feeding

Expressions of *CYP9Q1*, *TH*, and *IRS* mRNA in the brain (10 brains per group) were quantified using an SYBR Green real-time PCR method. According to the instructions of the Hieff^®^ qPCR SYBR^®^ Green Master Mix Kit, the reaction mix consisted of the following: master mix reagent: 5 µL; cDNA template: 0.2 µL; primer: 0.4 µL; ddH_2_O: 4.4 µL for reaction. *β-actin* was chosen as the optimal internal reference gene to normalize the expression levels between samples (Table 2).

### 4.9. Statistics

The Wilcoxon rank-sum test was used for statistical analysis, with *p*-values < 0.05 considered significant. The qPCR data were statistically analyzed using SPSS 8.0 (SPSS Science, IBM, Armonk, NY, USA) and GraphPad Prism 8.0 software. The data are presented as mean ± SD.

## Figures and Tables

**Table 1:** Summary of data quality control.

Sample	Raw Data	Valid Data	Valid Data (%)	Q20 (%)	Q30 (%)
F2-1	42,238,106	41,724,032	98.78%	97.26%	92.65%
F2-2	47,008,752	46,466,338	98.85%	97.54%	93.15%
F2-3	43,534,018	42,778,170	98.26%	97.64%	93.31%
H2-1	44,119,100	43,403,162	98.38%	97.44%	93.01%
H2-2	41,698,078	40,825,646	97.91%	97.36%	92.79%
H2-3	40,543,520	39,902,528	98.42%	97.32%	92.67%

Note: F2 represents sated after 2 h of starvation, H2 represents starved for 2 h.

**Table 2:** Primers used for Real-Time Quantitative PCR (qPCR).

Gene Name	Primer Sequences (5′ → 3′)	Product Size (bp)
*CYP9Q1*-F	GACCGTGACTGACATAGCCAATC	135
*CYP9Q1*-R	ATCTCCTCCTGAAGCCTCTGTTG	
*TH*-F	TGAGATACTGGACAGCGTGGAC	87
*TH*-R	TTGTTGACAGCGTTCGTGAGATG	
*IRS*-F	GTCGCATGGTGGCAGTAGTTG	143
*IRS*-R	TGGAATAGGCTCTTGCTGGTCTC	
*β-Actin*-F	CCTAGCACCATCCACCATGAA	87
*β-Actin*-R	GAAGCAAGAATTGACCCACCAA	

## Data Availability

The raw RNA-sequence data were deposited in the National Center for Biotechnology Information (NCBI) (accession numbers: PRJNA1181656). [National Center for Biotechnology Information (NCBI)] [https://www.ncbi.nlm.nih.gov/sra/, accessed on 4 November 2024] [PRJNA1181656].

## Supplementary Materials

The supporting information can be downloaded at: https://www.mdpi.com/article/10.3390/ijms252413550/s1.
