# Supplementary material for: CYP9Q1 Modulates Dopamine to Increase Sugar Responsiveness in Honeybees (Apis mellifera)
Source: Int J Mol Sci. 2024 Dec 18;25(24):13550. doi: 10.3390/ijms252413550 (PMC11678407; doi:10.3390/ijms252413550)
Supplement: Supplementary file 1 [file ijms-25-13550-s001.zip › ijms-3329503-supplementary.pdf]

Supplementary Material:

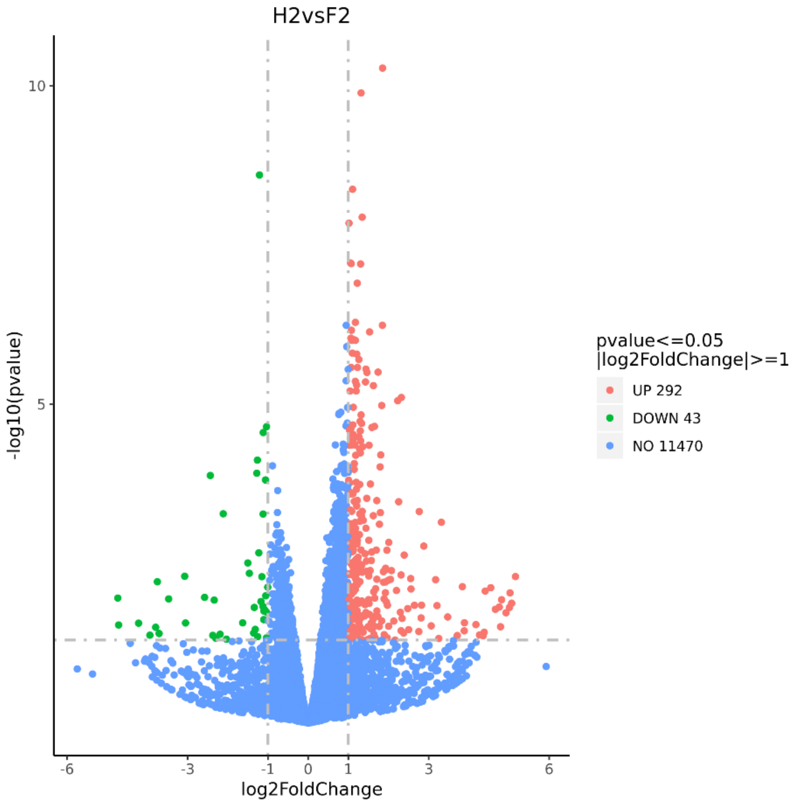

Supplementary Figure S1. A volcano plot of gene expression. Plots of differentially expressed genes (DEGs) are indicated in red and green.

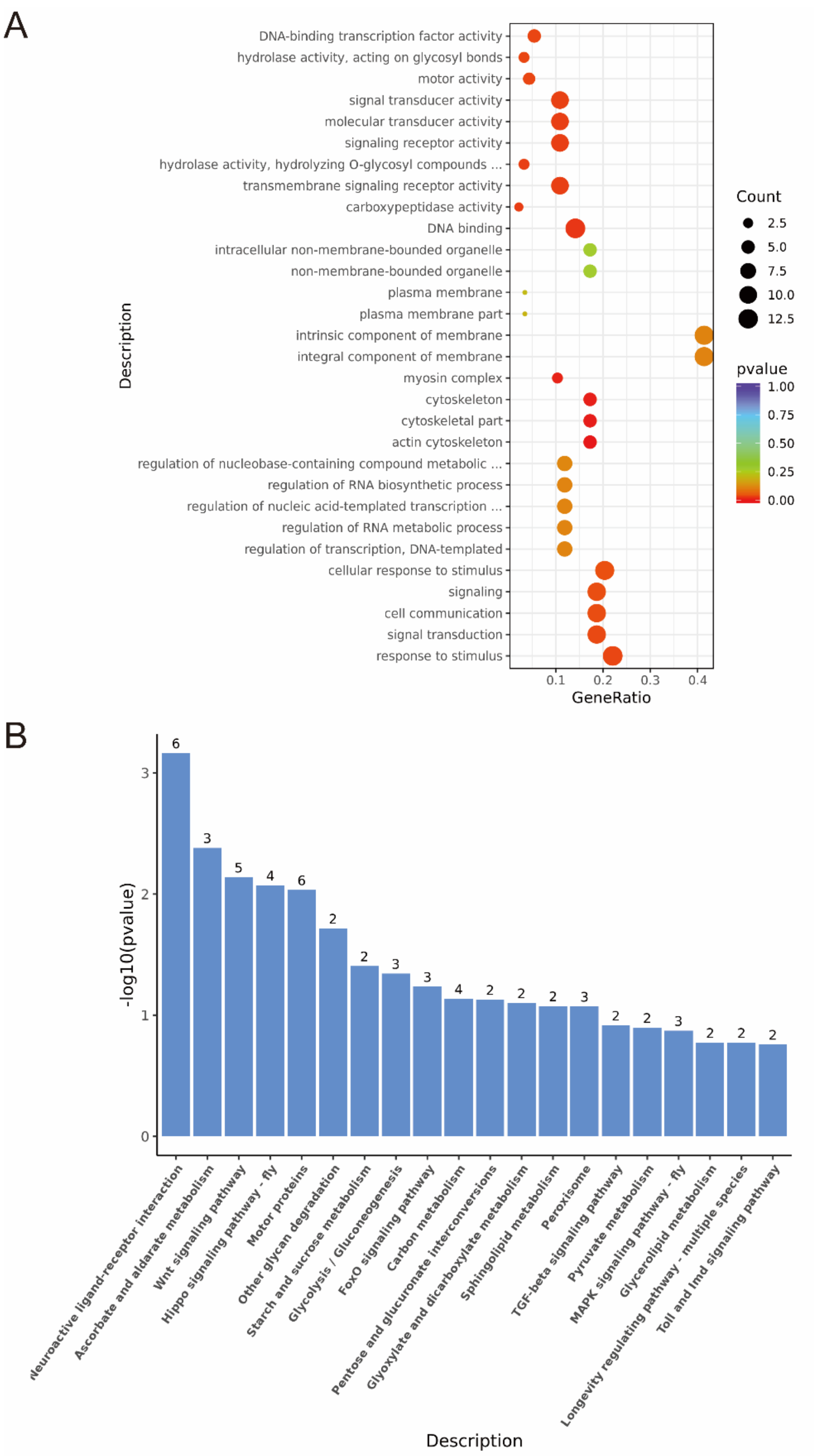

Supplementary Figure S2. (A) Gene ontology (GO) pathway enrichment analysis of the differentially expressed genes (DEGs). (B) Kyoto Encyclopedia of Genes and Genomes (KEGG) pathway enrichment result histogram. The abscissa is pathway. The vertical coordinate is the diagram of -log10 (*p*-value).

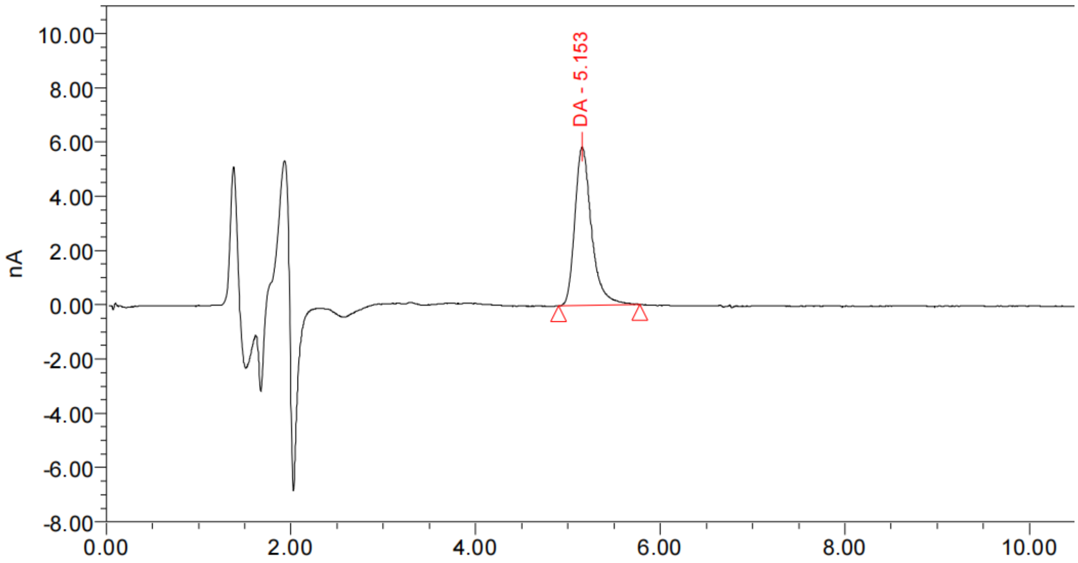

Supplementary Figure S3. High-performance liquid chromatography with electrochemical detection (HPLC-ECD) image of dopamine standard substance.

Supplementary Table S1. Primers used for Real-Time Quantitative PCR (qPCR).

Gene Name	Primer Sequences (5′→3′)	Product Size (bp)
LOC408807-F	TTTCATCAGGGATTGGGGCATAACC	135
LOC408807-R	TGAGGCAACGAGTCGGAGGAG	
LOC411184-F	CGTGAAGGCTGCGGCTGAAG	84
LOC411184-R	TCCTCCTCCTCCTCCTCTTCCTC	
LOC551176-F	CGGCGGCACCACCATGTATC	130
LOC551176-R	TCCACCGATCCACACCGAGTAC	
LOC100577669-F	CCGCAGGTTTATTACAGGCAGGAG	96
LOC100577669-R	CCTCTGATTACCGCTTCGCTTCG	
LOC724993-F	GCCCTAAATCTGACCCGAGTTTGG	110
LOC724993-R	GAACAACGGAGAGCAGTGAGGATG	
LOC113218812-F	GCCGTTTGTCCCACGACCATC	124
LOC113218812-R	ACGCACGACACCCTCCTTATCC	
LOC551369-F	CGGCGGCACCACCATGTATC	130
LOC551369-R	TCCACCGATCCACACCGAGTAC	
